# Hamartoma of the breast in a man: A rare case report

**DOI:** 10.1097/MD.0000000000018372

**Published:** 2019-12-16

**Authors:** Mengxin Li, Gu Lin, Wu You, Wang Zhen, Chengzhao Xu, Jinghui Hong, Du Ye, Song Dong

**Affiliations:** Department of Breast Surgery, The First Hospital of Jilin University, Changchun, Jilin Province, China.

**Keywords:** breast cancer, mammary hamartoma, man

## Abstract

**Rationale:**

Mammary hamartoma is a rare benign breast tumor, composed of ducts, lobules, fibers, and adipose tissue. We describe a mammary hamartoma in a man; this is the fourth case being reported in the literature.

**Patient concerns:**

A 30-year-old man presented with a 1-month history of a painless mass in his right breast.

**Diagnosis:**

Ultrasound imaging and mammography revealed a lesion, approximately 2.0 cm × 2.0 cm in size, in the right breast, which was considered to be either a lipomyoma or an adenoma fibrosum.

**Interventions:**

The mass was surgically resected. Pathological examination confirmed the diagnosis of mammary hamartoma.

**Outcomes:**

The patient was discharged from the hospital after surgery. There was no sign of reoccurrence during a 1-year follow-up period.

**Lessons:**

At present, mammary hamartoma is considered to be a benign lesion, usually treated by surgical resection. Some reports have suggested a possible association between a hamartoma and the development of breast malignancy. The pathology and biology of an association between a mammary hamartoma and malignancy have not been defined to date.

## 1 Introduction

Mammary hamartoma in men is a rare disease that accounts for 0.12% to 0.24% of all breast tumors and <4.8% of all breast benign tumors.^[1]^ We identified 3 previous case reports regarding mammary hamartoma in men.^[2–4]^ The clinical diagnosis of hamartoma mainly depends on the pathological examination and, thus, is influenced by the detection method used and the experience of the pathologist. Therefore, the true incidence rate of hamartoma in men might be higher than is currently reported. Mammary hamartoma also presents with unique imaging features^[5]^ which can help clinicians in improving the preoperative diagnosis rate of mammary hamartoma and to distinguish malignant components. Herein, we report on a very rare case of mammary hamartoma in a male patient, focusing on the clinical and pathological characteristics for diagnosis and treatment.

## 2 Case report

We received approval from the Institutional Review Board of Jilin University First Hospital, Changchun, Jilin, China for the publication of this report; the patient also provided informed consent for the publication of this case report.

**Figure 1:**
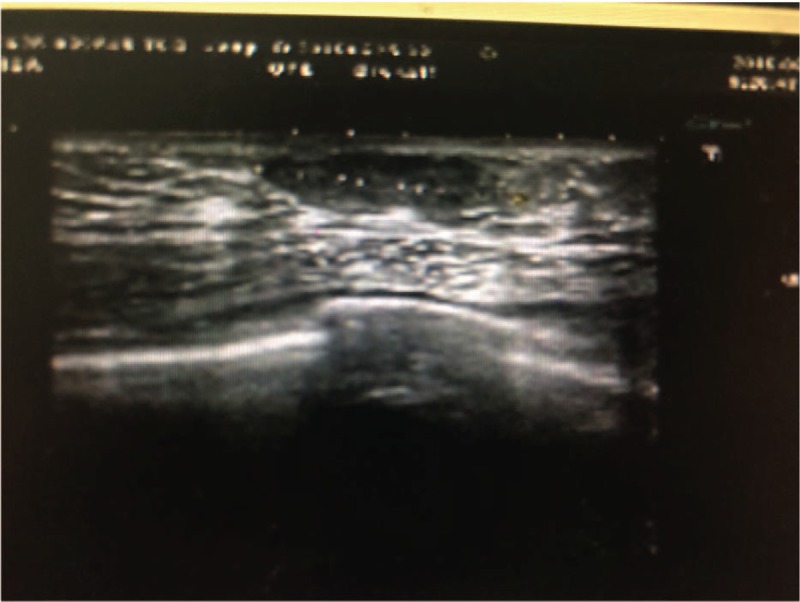
Ultrasound image, showing a well-encapsulated lesion.

**Figure 2:**
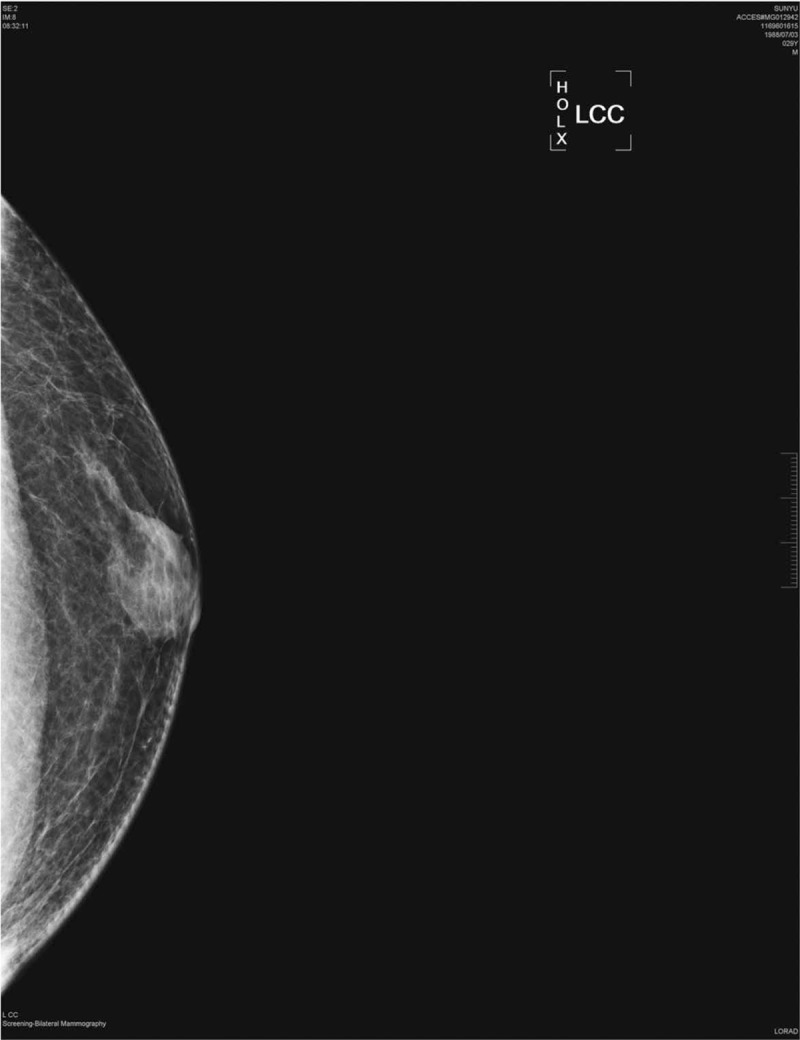
A mammogram, showing a well-circumscribed mass, of mixed density.

A 30-year-old man accidentally discovered a painless, quail egg-sized, mass in the lateral upper quadrant of the right breast, next to the nipple in February 2018. The patient did not initially seek medical consultation. In March 2018, the patient detected a painless enlargement of the mass and sought medical consultation at his local hospital. A breast ultrasonography examination revealed a mass, 23.7 mm × 7.5 mm in diameter (Fig. 1). No abnormality was identified in the left breast. Mammography revealed a well-circumscribed mass of mixed density in the right breast, with no evidence of invasion of the ipsilateral axillary nodes (Fig. 2). The patient refused to undergo fine needle aspiration cytology examination. The provisional clinical diagnosis was a lipomyoma or adenoma fibrosum.

**Figure 3:**
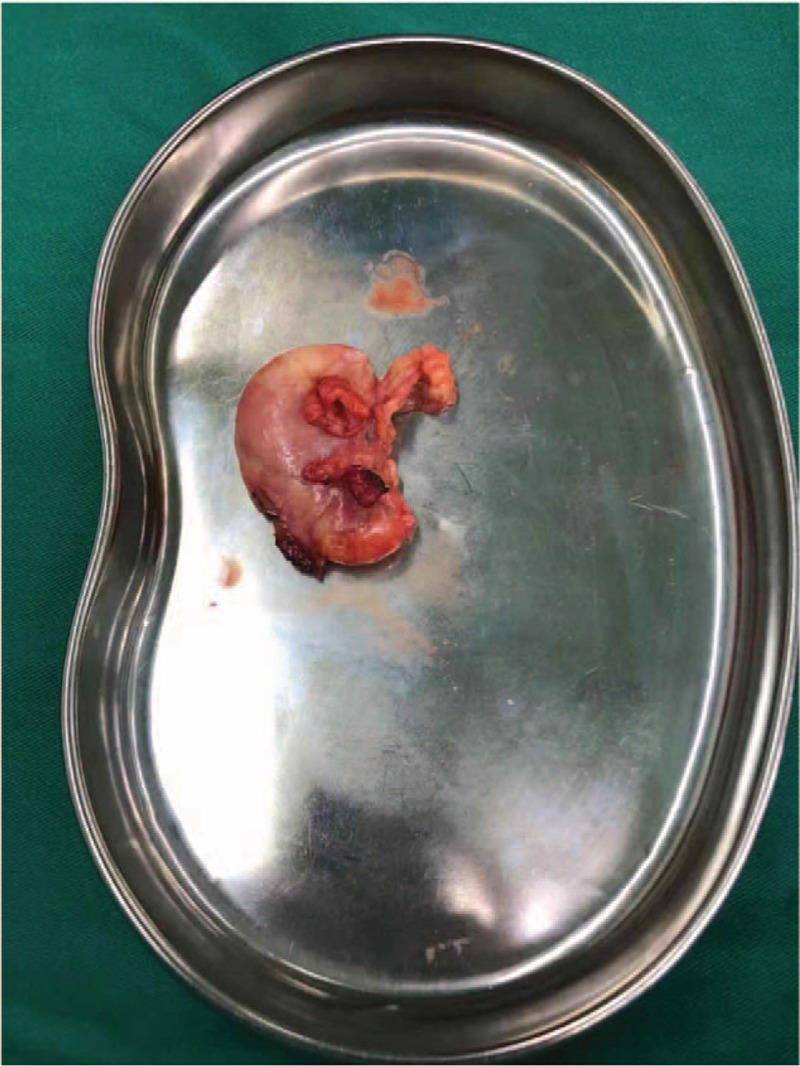
Gross and microscopic appearance of the fibroadenolipoma. The lesion is well-circumscribed and encapsulated, with gray-yellow coloring, resembling a fibroadenoma.

**Figure 4:**
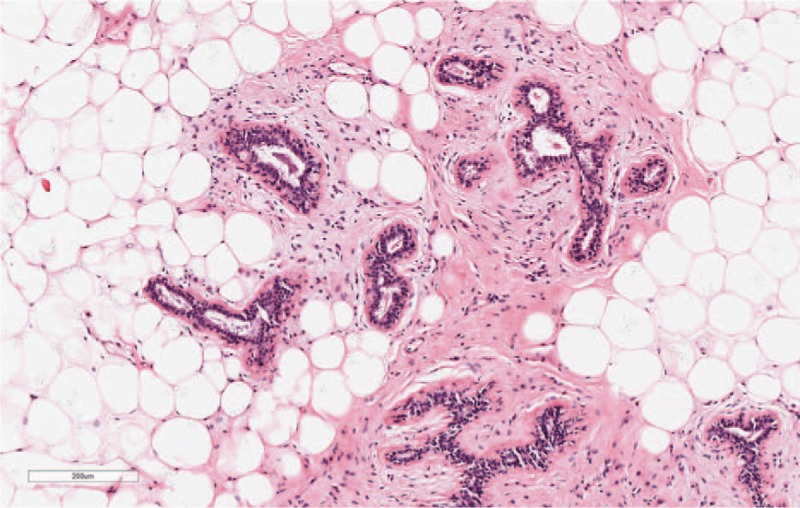
Hematoxylin and eosin stained mammary glandular tissue, showing mature adipocytes in hyalinized fibrous tissue (magnification, ×10).

The patient was referred to our hospital for further assessment, surgical management, and treatment. The patient's history was reviewed. We noted the following characteristics: current smoker (with a long history of smoking); no alcohol consumption; no history of trauma to the region; no history of prior surgery or radiation exposure of the region; and no personal or significant family history of cancer. With no important history identified, the clinical diagnosis of a hamartoma was established. The physical examination revealed a soft, mobile, painless mass located in the right breast, measuring approximately 2 cm in diameter, with no abnormality identified in the left breast. On March 27, 2018, the patient underwent surgical resection of the mass at our hospital. Gross examination of the resected mass revealed an oval, well-defined, and encapsulated mass, with gray-yellow coloring and a smooth margin (Fig. 3). The nodule was very soft and had a fibrotic envelope. Pathological examination confirmed the diagnosis of mammary hamartoma (Fig. 4). The patient recovered well after surgery, without complications and the need for further interventions. The patient was discharged from the hospital on postoperative day 7, April 4, 2018. The patient was followed-up every 6 months, with no sign of recurrence over a period of observation of 1 year.

## 3 Discussion

In 1971, Arrigoni et al^[6]^ first proposed the name “mammary hamartoma” and defined it as a well-defined mass formed by abnormal cells intermixed with normal mammary tissue. Most patients with mammary hamartoma are women, typically between the age of 33.5 and 66.5 years, with an average mass size of 5.0 cm.^[7]^ To date, only 3 cases of mammary hamartoma in men have been published. The first case^[2]^ involved a 36-year-old man presenting with a slowly growing mass in his left breast, with no evidence of axillary lymphadenopathy. The diagnosis of myoid hamartoma was confirmed after pathological examination. More recently, a case of hamartoma was reported in a 13-year-old boy, in whom the tumor extended into the thoracic cavity.^[4]^ Malignancy was considered and the mass was surgically resected, including 2 adjacent ribs. The diagnosis of hamartoma was confirmed on pathological examination. There is no documented recurrence of the mass in this patient. Due to the unique features of hamartomas, and of hamartomas in men, in particular, there is currently no standard treatment.

A hamartoma is usually a solitary, soft, round or oval, lesion, with an envelope, and is generally not tender.^[8]^ The growth of most mammary hamartomas is relatively slow. Patients with small tumors usually have no apparent clinical symptoms, and the hamartoma is identified incidentally on physical examination.^[9]^ However, some hamartomas can increase significantly in size over a short time,^[1]^ most commonly in pregnant and lactating women, indicating that hormones and endocrine factors may play a role in the development of these tumors.^[10]^ In men, there is a need to specifically differentiate a mammary hamartoma from male mammary development.^[11]^

Mammary hamartomas have specific imaging characteristics, which can greatly aid with the preoperative diagnosis. Moreover, recent studies have also reported on the unique features of nasal hamartoma^[12]^ and endobronchial hamartoma^[13]^ on radiological examination, with the diagnosis confirmed on pathological examination. Therefore, with more evidence, imaging may serve as a useful diagnostic tool to avoid unnecessary surgery. Approximately 94% of mammary hamartomas present with specific characteristics on mammography,^[14]^ namely as a round or oval mass, with uneven central density, and a smooth edge, accompanied by a transparent ring (consisting of a halo of fat).^[15–17]^ Calcification within a hamartoma is relatively rare. When present, massive calcification is considered to be benign, while small sites of silty calcification are usually associated with malignancy. Besides, the presence of micro-calcification shadow, a burr sign and lobulated tumor within the fat halo, on mammography, is also indicative of a possible malignancy.^[5]^ On ultrasound imaging, a mammary hamartoma presents as an oval or quasi-circular mass, with clear boundaries, parallel to the epidermis; an uneven echo structure, and mixed hypoechoic and hyperechoic tissue areas, with the majority of lesions having an intact capsule. Elastography can improve the identification of a hamartoma, based on the measured hardness of adipose tissue, glands, and fibrous connective tissue, which can aid in defining the boundary of the tumor.^[5]^ A myohamartoma reveals specific features on high-density T1-weighted magnetic resonance images, with a low-density shadow on T2-weighted images, although the specificity of these image-based findings has not been fully evaluated.^[18,19]^

A mammary hamartoma is composed of the same elements as normal mammary tissue and, thus, is difficult for pathologists to diagnose. Lumpectomy provides a curative treatment. Maintaining the integrity of the capsule during resection could improve the rate of pathological diagnosis. For rare types of hamartomas, such as myohamartomas, immunohistochemistry plays an important role in the diagnosis, with positive expression of specific markers (including smooth muscle actin, vimentin, and desmin) being strongly associated with the presence of spindle cells, and expression of s-100 and cytokeratin being negatively associated. Although the tumor is histologically benign and often painless, a hamartoma may develop to be quite large if local excision is not performed in a timely manner.^[20]^

Malignancy originating from a hamartoma is extremely rare, but is possible as these tumors contain epithelial tissue. As an example is the case of a 70-year-old woman treated for a mammary hamartoma, who presented with mammogram changes around the mass 6 years later and was diagnosed with invasive ductal carcinoma.^[21]^ The development of lobular carcinoma and invasive carcinoma has also been reported.^[21,22]^ However, it is unclear if malignancy associated with a hamartoma results from a transformation of hamartoma tissue or if the malignant cells extend from hamartoma tissue. Therefore, the relationship between a hamartoma and breast cancer needs further discussion.

Mammary hamartoma in men is rare, with the underlying pathology and biology remaining to be clearly defined. In addition, as men typically pay less attention to palpable masses within the breast tissue, it is commonly noted that lesions may grow substantively before medical attention is sought. As such, hamartoma in men tend to be detected later than in women, requiring differentiation from a malignant lesion. Our case report adds to the body of knowledge on this rare condition, with the accumulation of case reports being important to improve the diagnostic criteria, based on non-invasive techniques, and to establish, in time, a standard of care for this condition.

## Acknowledgments

The authors are grateful to the surgical department of the First Hospital of Jilin University for providing the patient's data.

## Author contributions

**Conceptualization:** Song Dong.

**Data curation:** Gu Lin.

**Formal analysis:** Wu You.

**Investigation:** Wang Zhen, Chengzhao Xu.

**Resources:** Du Ye.

**Project administration**: Song Dong

**Software:** Jinghui Hong.

**Supervision:** Song Dong.

**Writing – original draft:** Mengxin Li.

**Writing – review & editing:** Mengxin Li.

Mengxin Li orcid: 0000-0002-3071-1669.
